# Factors Associated with the Prevalence of Breastfeeding Periods in a Cohort of Spanish Primiparous Pregnant Women

**DOI:** 10.3390/nu16234086

**Published:** 2024-11-27

**Authors:** Cristina Bouzas, Eva Pilar López-García, Mercedes Sánchez-Martínez, Josep A. Tur, Rosario Pastor

**Affiliations:** 1 CIBER Fisiopatología de la Obesidad y Nutrición (CIBEROBN), Instituto de Salud Carlos III (ISCIII), 28029 Madrid, Spain; 2 Research Group on Community Nutrition & Oxidative Stress, University of Balearic Islands-IUNICS, IDISBA & CIBEROBN, Guillem Colom Bldg, Campus, 07122 Palma de Mallorca, Spain; rosario.pastor@ucavila.es; 3 Health Research Institute of the Balearic Islands (IdISBa), 07120 Palma de Mallorca, Spain; 4 Faculty of Health Sciences, Catholic University of Avila Santa Teresa de Jesus, 05005 Avila, Spain

**Keywords:** breastfeeding, primiparous pregnant women, prolactin, vitamin B_12_, folic acid, transferrin, informal caregiver, health education

## Abstract

Aims: To assess factors associated with the prevalence of breastfeeding in a cohort of Spanish primiparous pregnant women to whom a health education program was applied. Methods: Prospective, descriptive, and inferential cohort analysis with data obtained from the beginning of pregnancy to the child’s year. Prevalence of breastfeeding was classified into periods or categories: (1) “non-breastfeeding”; (2) “breastfeeding until 6 months”; (3) “breastfeeding up to 12 months”. The sample was 288 primiparous pregnant women divided into two groups of equal size using as a matching strategy whether they attended the health education sessions with or without an informal caregiver (mother, partner, sister): group A (primiparous women who attended the sessions without an informal caregiver; n = 144) and group B (primiparous women who attended the sessions with an informal caregiver; n = 144). Results: The best-associated variables with the prevalence of breastfeeding were age, vitamin B_12_ variation (postintervention—preintervention), and informal caregiver intervention. This association was statistically significant for breastfeeding up to 12 months of age compared to non-breastfeeding (age (*p* = 0.007); vitamin B_12_ variation (*p* = 0.010); caregiver intervention (*p* = 0.008)). The younger the breastfeeding woman, the greater the probability of breastfeeding up to 12 months (β: −0.246 (0.91); OR: 0.782 (0.654–0.935)). The greater the increase in vitamin B_12_ after the educational intervention program, the greater the probability of breastfeeding up to 12 months (β: 0.007 (0.003); OR: 1.007 (1.002–1.012)). The absence of an informal caregiver decreased the likelihood that breastfeeding would be maintained until the child’s 12 months (β: −0.734 (1.024); OR: 0.065 (0.009–0.483)). Conclusions: The prevalence of breastfeeding up to 12 months, in a cohort of Spanish primiparous women, increased the higher increase in vitamin B_12_ levels after applying an educational intervention program to promote breastfeeding. The prevalence of breastfeeding up to 12 months also increased with the presence of the informal caregiver and in young women.

## 1. Introduction

Despite well-documented evidence on the importance of breastfeeding and its benefits, both for newborns and mothers [1,2,3], prevalence rates do not reach adequate levels. This is a common aspect in Western countries, where the social and health levels should report higher figures [4,5]. In Spain, according to data from the National Institute of Statistics (NIE), the rate of exclusive breastfeeding during the first six months of life in 2023 was 24.72% [6].

The World Health Organization (WHO) recommended starting breastfeeding in the first 24 h of the newborn’s life, and maintaining it exclusively for the following six months, continuing until the age of two with complementary feeding [7]. These recommendations are in accordance with the involved Scientific Societies [8,9,10]. However, it was shown that these recommendations were not met. Thus, the United Nations International Children’s Emergency Fund (UNICEF) in 2018 reported that only two of five newborns worldwide were breastfed during the first hour of life [11]. In Spain, according to NIE data, in the first six and a half months of the newborn’s life, there was a high rate of breastfeeding, but this rate decreased considerably at three months of age, showing an important reduction at six months of age [6].

Numerous factors, both maternal and infant, related to the initiation and continuity of breastfeeding were analyzed. These factors included pre-pregnancy obesity, smoking, delivery modality, and breastfeeding education [12]. However, heterogeneity in the studies’ design was often high, making as difficult to discern patterns that could lead to successful interventions [13,14].

Therefore, it is important to assess the factors associated with the prevalence of breastfeeding, at least until twelve months of age. An understanding of the relationships between demographic, socioeconomic, and lifestyle factors will allow health interventions to increase breastfeeding adherence.

In Spain, the LAyDI study published in 2023 [15] was carried out for two years on a cohort of newborns, and aimed to know the prevalence of breastfeeding, as well as the sociocultural and perinatal factors affecting its initiation and maintenance. However, this study did not follow women throughout pregnancy, nor assessed factors such as the effectiveness of the influence of informal caregivers.

The aim of the current study was to assess factors associated with the prevalence of breastfeeding in a cohort of Spanish primiparous pregnant women, to whom a health education program was applied.

## 2. Methods

### 2.1. Study Design

The breastfeeding prevalence rate was measured before and after an intervention by a health education program on a prospective, descriptive, and inferential cohort of primiparous pregnant women exposed and not exposed to the presence or not of primary caregivers from the beginning of pregnancy to the child’s first year of life.

### 2.2. Description of Random Variables

The prevalence rate of breastfeeding was classified into the following periods or categories: (1) “non-breastfeeding”; (2) “breastfeeding until 6 months”; (3) “breastfeeding up to 12 months”. Women who breastfed for less than 6 months were included in the first group “breastfeeding up to 6 months” and those who breastfed for more than 6 months and less than 12 months were included in the group “breastfeeding up to 12 months”.

The following variables were measured as factors that could potentially be associated with the prevalence of breastfeeding periods: (1) sociodemographic factors: age (years); nationality (i: Spanish/European; ii: South American; iii: others); level of education (i: primary; ii: secondary; iii: higher education); work (Yes/No); couple (Yes/No); couple’s level of education (i: primary; ii: secondary; iii: higher education); contraceptives (Yes/No); smoking habit (Yes/No); alcohol consumption (Yes/No); family history of breastfeeding (Yes/No); food supplements (Yes/No); informal caregiver (Yes/No); informal caregiver type (I: none; II: mother; iii: partner; iv: sibling); breastfeeding in the first hour postpartum (Yes/No); (2) biochemical parameters (preintervention and postintervention education): prolactin (ng/mL); folic acid (ng/mL); vitamin B_12_ (pg/mL); transferrin (mg/dL); and (3) body weight (kg) (i: first trimester of pregnancy; ii: second trimester; iii: postpartum).

### 2.3. Participants, Recruitment, Randomization, and Ethics

To select the participant women in the study, medical records of the cohort of primiparous pregnant women were assessed under the coding of the clinical process of pregnancy in the digital registry Medora@ [16,17]. The sample was a cohort of 288 primiparous pregnant women belonging to a Primary Health Center in central Spain. 

For the calculation of the necessary sample size, the value of breastfeeding was considered as the main study parameter (SD = 4, difference to be detected between groups in the mean changes = 1.5), in a bilateral contrast, resulting in a sample size of 125 subjects in group A and 125 subjects in group B. An alpha risk of 0.05 and a statistical power greater than 0.8 were considered. A follow-up loss rate of 10% was estimated.

The final sample consisted of 288 primiparous pregnant women, who were divided into two groups of equal size, using as a matching strategy whether they attended the health education sessions with or without an informal caregiver (mother, partner, sister): group A (primiparous women who attended the sessions without an informal caregiver; n = 144) and group B (primiparous women who attended the sessions with an informal caregiver; n = 144). To determine the assignment to group A or B, the informal caregiver was identified by the pregnant woman during the pregnancy and puerperium process. The objective of this matching strategy was to establish the influence of exposure to a factor (in this case the inclusion of the informal caregiver in health education) on the results of the health education program, referring to breastfeeding rates, as well as on the contribution of nutrients of special relevance in pregnancy and breastfeeding.

The inclusion criteria were: (1) primiparous pregnant women with or without an identified or referral informal caregiver; (2) primiparous women belonging to the Primary Health Centre with a diagnosis of normal pregnancy recorded in the medical record; (3) women treated at the Primary Health Centre from December 2020 to October 2021; and (4) primary caregiver identified by the pregnant woman during the pregnancy and puerperium process, capable of granting informed consent.

The exclusion criteria were as follows: (1) primiparous women with a serious clinical situation, complicated or pathological pregnancy; (2) primiparous women diagnosed with COVID-19 infection during pregnancy, three months before becoming pregnant or during breastfeeding (so as not to obtain interference in the analytical parameters); and (3) primiparous women in contact or symptoms compatible with COVID-19 infection without occupational confirmation of infection.

The study protocols followed the Declaration of Helsinki ethical standards. Clearance was obtained from the Ethics Committee for Clinical Research of East Valladolid Health Area (ref. PI 20-2068 AP COVID-19, 25 June 2020), and following the Agreements and Standards established in the Spanish legislation related to biomedical research, data protection, and cough of personal nature and bioethics. All participating institutions approved the study protocol and procedures according to the Declaration of Helsinki’s ethical standards. All participants provided written informed consent.

### 2.4. Educational Intervention Program

**Figure 1:**
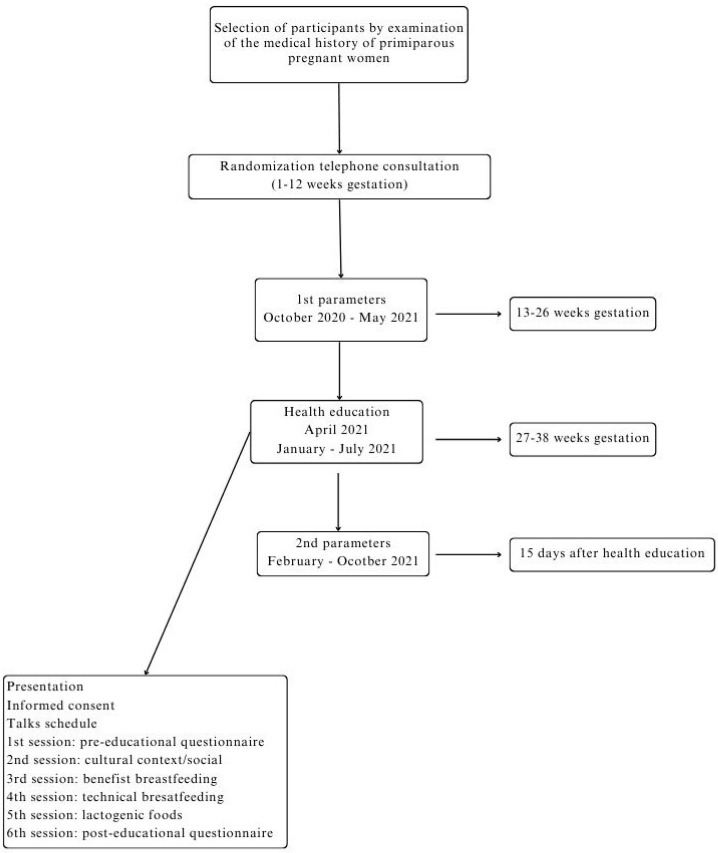
Flowchart of recruitment and interventions into two groups.

Figure 1 shows the flow chart of the recruitment and the intervention carried out in two groups (A and B).

The intervention was planned in three stages:

First stage: The midwives were contacted and asked for their collaboration to select the sample, and contact and recruit the pregnant women, and primary informal caregivers. The selection of the study population was carried out by reviewing the nominal and numeral records of clinical processes of pregnancy in the digital medical database program (Medora@) [18], with the agreement of the Primary Health Care Management (Valladolid, Spain), to access the medical record for research purposes. The pregnant women and informal caregivers were contacted by telephone. They were interviewed and informed about the project. After the agreement to participate in the study, both pregnant women and informal caregivers signed the informed consent. Blood parameters (prolactin, folic acid, vitamin B_12_, and transferrin) of pregnant women were measured in the second trimester of pregnancy (13–26 weeks).

Second stage: A double intervention of health education (27–38 weeks of pregnancy) was carried out in the two groups (with an informal caregiver and without an informal caregiver) as previously defined. The two groups (A and B) received health education talks on breastfeeding, where the benefits for the mother, the baby, and the family were explained [19], including lactogenic techniques and foods. The level of knowledge of the two groups before and after the educational intervention was compared, through previously validated pre-educational and post-educational questionnaires [20]. These questionnaires had two main contents to be developed by different questions: knowledge of healthy foods to consume during pregnancy to increase milk production, lactogenic foods, and the importance of family support. All participants were followed up until the first postpartum visit, and then the breastfeeding index was assessed by follow-up until the child was 12 months old. To design the content of the education talks, a group didactic program was carried out favoring the participation of pregnant women. An important aspect used in the intervention was the work in the cognitive, affective-attitudinal, and psychomotor spheres [21]. Emphasis was placed on oral, gestural, visual, and human support, which served to transmit an idea, stimulate, motivate, and help change [22]. Moreover, the effective transmission of messages was ensured to achieve a reaction, response, or impact.

Health education talks: (1) first session: completion of the pre-educational questionnaire and staging of statistical data/prevalence of breastfeeding at the global, national, and regional levels; (2) second session: presentation of historical evolution, cultural, and/or social context; (3) third session: benefits of breastfeeding for the baby, the mother, and the family; (4) fourth session: technique and duration of breastfeeding; (5) fifth session: adequate nutrition during pregnancy (lactogenic foods); and (6) sixth session: completion of the post-educational questionnaire.

Third stage: Blood parameters were analyzed in the third trimester of pregnancy (38–40 weeks), 15 days after the application of the health education program.

### 2.5. Blood Parameter Measurement

To correctly collect the blood samples (to measure prolactin, folic acid, vitamin B_12_, and transferrin levels), each participant was instructed on the ideal conditions to follow: do not practice exercise two hours before the collection of the sample, be relaxed 30 min before, avoid stressful situations, avoid a diet rich in proteins and fats the day before the collection of the sample, fast for 8 to 10 h, and do not take medications that can raise or decrease the value [5,6]. The extraction was performed by venipuncture in veins located in the antecubital area with a 21 G wing nut with an adapter BDVacutainer Safety Lok@, a 2.5 cm × 45 cm latex venous compressor, and a tube with separator gel (yellow cap) for samples of prolactin, folic acid, vitamin B_12_, and transferrin. This tube was anticoagulant-free and contained serum separator gel and cellular elements, which kept the serum stable for more than 48 h, and there was no obvious change in its biochemical characteristics and chemical compositions [23]. For hematological and biochemical determinations, blood was extracted in a reference hospital laboratory (University Clinical Hospital, Valladolid, Spain). The necessary material and human resources were counted, without interfering in the performance of other studies or tasks that are usually entrusted to nurses.

Prolactin is the hormone responsible for the production, supply and maintenance of milk during pregnancy and lactation. Its levels begin to increase more markedly during the second month of pregnancy. If the mother did not breastfeed the newborn, prolactin levels returned to normal after delivery. When a woman was breastfeeding, milk supply was also influenced by the newborn’s sucking, as the more she sucked, the more milk was produced [24].

Vitamin B_12_ and folic acid are closely related metabolically and are involved in different processes in the body, such as the maturation and division of cells and the myelination of the nervous system. Therefore, these vitamins are essential for the normal growth and development of the fetus during pregnancy and the first years of life [25].

Transferrin is another important parameter during these periods, as it allows the normalized formation of red blood cells and hemoglobin [26].

### 2.6. Statistics

SPSS statistical software package version 27.0 (SPSS Inc., Chicago, IL, USA) was used to perform statistical analysis. The Kolmogorov–Smirnov test was applied to analyze the distribution of the variables, observing that they did not follow a normal distribution.Categorical variables are shown as sample size and percentage, and continuous variables as median and interquartile range. Differences between breastfeeding groups for ordinal variables were tested by Kruskal–Wallis test (grouping variable: breastfeeding categories) and Games–Howel post hoc. Differences in prevalence across groups were examined using χ^2^.

Changes in biochemical parameters during the educational intervention and changes in body weight were analyzed by two-way repeated measures, Quade’s non-parametric ANCOVA adjusted age (intra-subject factor: “time”; inter-subject factor: “breastfeeding”). The pairwise comparison tables of the intra-subject factors time and time × breastfeeding were adjusted using Bonferroni. Levene’s test was used to assume equality of variances (*p* > 0.05) and apply Tukey’s post hoc analysis between the levels of the inter-subject factor “breastfeeding” assuming equality of variances.

To analyze the possible factors associated with breastfeeding periods, a multinomial logistic regression was performed. The data entry method was backward. The methods of elimination were the likelihood ratio and Wald’s criterion. The dependent variable was breastfeeding groups. In the first model, independent variables were considered those in which statistically significant differences were observed in the tests previously described in this section (factors: categorical variables; covariates; continuous variables). 

## 3. Results

### 3.1. Sociodemographic Characteristics of the Participants

Table 1 shows the sociodemographic characteristics of the participants, as well as information on informal caregivers and breastfeeding in the first postpartum hour. 

Differences were observed between groups for smoking habit (*p* = 0.026) and educational level (*p* = 0.004). Differences between groups were also observed in age of breastfeeding (*p* = 0.029). Post hoc analysis showed that there were only significant differences in the age of breastfeeding between the categories “non-breastfeeding” and “breastfeeding up to 12 months” (*p* = 0.008).

Differences were found between the breastfeeding categories (*p* < 0.001 for both variables). A higher prevalence of caregiver and breastfeeding intervention in the first hour postpartum was observed in the breastfeeding group up to 12 months than in the other groups. Differences were also observed between groups for informal caregivers (*p* < 0.001).

### 3.2. Changes in Biochemical Parameters During the Health Education Intervention and Body Weight

The changes in biochemical parameters during the educational intervention, and the changes in body weight of women during pregnancy and between the first trimester and postpartum, according to the different breastfeeding groups, are available in Table 2. 

No differences were observed in the change of biochemical parameters over time within the same breastfeeding group. No significant differences were observed in the body weight of participants between the first trimester of pregnancy and postpartum.

Differences were observed before the intervention between the group that did not breastfeed and the group that gave up to 12 months for prolactin (mean difference (non-breastfeeding—lactation 12 months) (95% CI); *p*-value): (16.914 (2.825–31.002); *p* = 0.013), vitamin B_12_ (mean difference (non-breastfeeding—lactation 12 months) (95% CI); *p*-value): (−23.659 (−37.059–10.258); *p* < 0.01), and transferrin (mean difference (non-breastfeeding—lactation 12 months) (95% CI); *p*-value): (20.994 (7.239–34.749); *p* = 0.001).

Differences were also observed at baseline between the non-breastfeeding and 6-month group for vitamin B_12_ (mean difference (non-breastfeeding—lactation 6 months) (95% CI), *p*-value): (−17.848 (−35.600–−0.096); *p* = 0.048).

In the postintervention timeline, differences were found between the “non-breastfeeding” and “breastfeeding up to 12 months” groups for prolactin (mean difference (non-breastfeeding—lactation 12 months) (95% CI); *p*-value): (−31.002 (−43.360–−18.644); *p* < 0.001), folic acid (mean difference (non-breastfeeding—lactation 12 months) (95% CI; *p*-value)): (−27.073 (−40.186–−13.960); *p* < 0.001), vitamin B_12_ (mean difference (non-breastfeeding—lactation 12 months) (95% CI); *p*-value): (−35.706 (−47.077–−24.336); *p* < 0.001), and transferrin (mean difference (non-lactation—lactation 12 months) (95% CI); *p*-value): (−19.916 (−33.389–−6.443); *p* = 0.002).

In this same timeline, differences were observed for vitamin B_12_ in the groups “non-lactation” and “lactation up to 6 months (mean difference (non-breastfeeding—lactation 12 months) (95% CI); *p*-value): (−17.716 (−32.776–−2.653); *p* = 0.015) and the groups “breastfeeding up to 6 months” and “breastfeeding up to 12 months” (mean difference (non-breastfeeding—breastfeeding 12 months) (95% CI); *p*-value): (−17.991 (−33.997–−1.985); *p* = 0.022).

Differences in transferrin levels were also found between the group that did not breastfeed and the group that did breastfeed up to 6 months (mean difference (non-breastfeeding—lactation 12 months) (95% CI); *p*-value): (−20.273 (−38.120–−2.425); *p* = 0.020). 

No differences were found between the groups for body weight variations.

During the educational intervention, significant differences were found for folic acid (*p* = 0.026) and vitamin B_12_ (*p* < 0.01) between breastfeeding groups. Post hoc analysis showed differences between “non-lactation” and “lactation up to 12 months” groups for folic acid (mean difference (non-lactation—lactation 12 months) (95% CI); *p*-value): (−9.807 (−18.611–−1.003); *p* = 0.025), whereas for vitamin B_12_ differences between “non-lactation” and “lactation 6 months” groups (mean difference (non-lactation—lactation 6 months (95% CI); *p*-value) were found: (−17.782 (−30.381–−5.182); *p* = 0.03) and for “non-lactation” and “lactation up to 12 months” groups (mean difference (non-lactation-lactation 12 months) (95% CI); *p*-value): (−29.682 (−39.194–−20.171); *p* = 0.01).

### 3.3. Factors Associated with Breastfeeding Periods

Table 3 shows the results of the final multinomial logistic regression, including the predictor variables that provided a better fit of the model. The model was significant (Chi-square = 63.987; *p* < 0.01) and explained 60% (R^2^ = 0.600) of the dependent variable (breastfeeding periods).

The variables that best explained the prevalence of breastfeeding and, therefore, the greatest association with breastfeeding were age, vitamin B_12_ variation (postintervention—preintervention), and informal caregiver intervention. This association was significant for the prevalence of breastfeeding up to 12 months of age compared to the non-breastfeeding group (age (*p* = 0.007); vitamin B_12_ variation (*p* = 0.010); caregiver intervention (*p* = 0.008)). Thus, the younger the breastfeeding woman, the greater the probability of breastfeeding prevalence up to 12 months (β: −0.246 (0.91); OR: 0.782 (0.654–0.935)). The greater the increase in vitamin B_12_ after the educational intervention program, the greater the probability of breastfeeding up to 12 months (β: 0.007 (0.003); OR: 1.007 (1.002–1.012)). The absence of an informal caregiver decreased the likelihood that breastfeeding would be maintained until the child’s 12 months (β: −0.734 (1.024); OR: 0.065 (0.009–0.483)).

## 4. Discussion

The overall prevalence of breastfeeding in the current studied cohort was 46.6%, which was slightly higher than the reported by the LAyDI study [15], in which 40.1% of 12-month-old children continued breastfeeding. In the current study, the prevalence of breastfeeding by periods of women who breastfed their children, only 31.3% gave up at 6 months, while 68.7% continued until 12 months. The current reported prevalence up to 6 months was slightly lower than those obtained in the LAyDI study, which was 35.2%. Other studies carried out in Spain at the regional level reported 54.3% and 27.8% (Aragón) [27], 48.4%, and 20.6% (Basque Country) [28] of the prevalence of total breastfeeding at 6 months and 12 months, respectively.

Buckman et al. [29] compared the prevalence and factors associated with breastfeeding in primiparous versus multiparous women and reported that primiparous women were more likely to breastfeed than multiparous women but having a shorter average duration of breastfeeding. This could explain the high percentage of women in the current cohort who did not breastfeed (53.5%), since they were all primiparous. According to Hackman et al. [30], first-time mothers may be overly optimistic about their breastfeeding goals, especially those who plan to extend breastfeeding by more than 6 months, without understanding the associated challenges. This did not occur in the current cohort, since a higher percentage of the women who breastfed their children prolonged breastfeeding for up to 12 months compared to those who breastfed up to 6 months.

The current results showed a significant association between maternal age and breastfeeding prevalence. It was observed that the younger the mother’s age, the greater the probability of breastfeeding prevalence up to 12 months of the child’s age. These results are consistent with those reported by McGowan et al. [31], which assessed the prevalence of breastfeeding in the U.S. and predictors of duration of breastfeeding for 24 or more months, using data from a nationally representative survey. Mothers over 30 years of age were less likely to breastfeed for 24 or more months. In the current study, within the 154 women who did not breastfeed, 66% were over 30 years old, while of the 28 women who breastfed up to 12 months, only 28.6% were over 30 years old.

The analysis of the variation of biochemical parameters during the educational intervention program for the promotion of breastfeeding reported significant differences between the breastfeeding categories for folic acid and vitamin B_12_. Regarding folic acid, the increase was greater in the group that breastfed up to 12 months compared to the group that did not breastfeed, while for vitamin B_12_ higher increases were observed both in the group that breastfed at 6 months and in the group that breastfed up to 12 months, than the group that did not breastfeed. The subsequent association analysis showed a significant association between the increase in B_12_ during the program and the breastfeeding group up to 12 months, compared to the non-breastfeeding group. In other words, the greater the increase in vitamin B_12_ levels after the health education program, the greater the probability of breastfeeding prevalence up to 12 months. These results may show the effectiveness of the program applied in terms of improving knowledge of foods rich in vitamin B_12_ and increasing its intake. In the study conducted by Bjørkevoll et al. [25], in healthy pregnant women and their babies in Norway, the authors observed that babies breastfed up to 3 and 6 months had a lower level of vitamin B_12_ compared to non-breastfed babies; however, the strength of these results may be limited by the low number of infants in the sample who were not breastfed until 3 and 6 months. Moreover, it is important to note that in this previous study, a health education program was not applied to women, and it did in the current study, a fact that supports our theory and could explain the difference in the results obtained in our study compared to those obtained by Bjørkevoll et al. [25].

Regarding the intake of food supplements, no differences were observed between the different breastfeeding groups; the significant association observed between the increase in B_12_ and the prevalence of breastfeeding up to 12 months may be due to the health education program itself, the presence of the informal caregiver in the sessions, and this intervention as support for breastfeeding women, as well as to differences between the levels of this nutrient between the different groups for each of the timelines or an interaction between these factors.

As already detailed in the current results, no differences observed between the timelines (postintervention—preintervention) may mean that the educational intervention program alone did not influence the results. However, differences were observed between the breastfeeding groups for both timelines separately, always in favor of the group that breastfed up to 12 months, although the multivariate analysis within time*group subjects did not show differences.

The current study showed that the presence of the informal caregiver increased the probability of breastfeeding prevalence up to 12 months. Therefore, the greater increase in B_12_ in the group that breastfed until 12 months could be explained mainly by the presence of the informal caregiver in the sessions of the current health education program. In other words, it may be due to an interaction between the program applied to pregnant women and the presence of the caregiver, although the differences between the levels of this B_12_ in the different groups for each line of health care must be considered.

There is enough published scientific evidence on the importance of health education during pregnancy and the postpartum period, and of the intervention of the informal caregiver. Thus, research carried out on pregnant women controlled in hospital outpatient clinics [32] concluded that educational support for mothers regarding breastfeeding both before and after childbirth increased the prevalence of breastfeeding up to six months of the newborn. Therefore, it is important to provide comprehensive and integrated care to caregivers, to manage the workload, both objective and subjective, on the care of pregnant women and on the subsequent breastfeeding period entails for them [33].

Both physical and emotional support from the woman’s primary caregiver is particularly effective in primiparous women with no prior experience. This support will significantly condition the adoption of healthy eating habits during pregnancy and the postpartum period, and may influence the prevalence of breastfeeding initiation and guarantee its success [34].

The results of a meta-analysis conducted by Cohen et al. [35] showed significant evidence that increased education and support for mothers or fathers during pregnancy or shortly after birth improved the initiation and continuation of breastfeeding. Attending prenatal breastfeeding classes likely provided women with strategies to cope with the challenges associated with the first few weeks of breastfeeding, such as breast engorgement, cracked nipples, perceived insufficient milk supply, etc. [36], as well as longer-term strategies, such as establishing breastfeeding routines or breast pumping [37]. A review by Gavine et al. [38] described and evaluated the effectiveness of different breastfeeding support interventions. These authors grouped the interventions into two different categories: “breastfeeding only”, which included interventions that only included breastfeeding support; and “breastfeeding plus”, in which breastfeeding support was part of a broader intervention that also aimed to provide other health benefits for the mother or the baby. Overall, the clinical trials included in this review showed that fewer women who received a “breastfeeding-only” intervention were likely to stop exclusively breastfeeding at all time points up to and including six months, although the evidence for these findings was moderate. There was not enough evidence of whether interventions of this kind could decrease the number of women who stopped breastfeeding at nine or twelve months. Women who received “breastfeeding plus” support did not show clear results on whether these interventions decreased the number of women who stop either breastfeeding or exclusive breastfeeding at different time points.

Regarding whom providing the support (professional or non-professional) or how it was provided (in person, digital technologies or combinations), there did not seem to be significant differences. In the current study, a program was applied that we could include in the second type described by Gavine et al. [38], and in it, the intervention of professionals and non-professionals (informal caregivers) was combined, showing a possible influence of the informal caregiver on the current results. Accordingly, support is a complex intervention that addresses a multifaceted challenge, and it should not be surprising if the results vary in different settings and studies. One initiative that showed higher rates of breastfeeding is the WHO’s “Baby-friendly Hospital Initiative” [39,40,41,42], which is a complex intervention that incorporates 10 steps for successful breastfeeding.

Different studies reported that the prevalence of breastfeeding decreased in women with obesity during the pregestational period and the breastfeeding period [35]. In the current study, no differences were observed between the different breastfeeding groups for body mass index (BMI), both pregestational and post-gestational, nor were there differences observed for body weight variation in any of the times analyzed (third trimester—first trimester; postpartum—first trimester). These results are probably due to the low percentage of women with obesity in the cohort analyzed, since only eight women had postpartum obesity and three had pregestational obesity, both measured as a function of BMI.

Several studies established a positive association between smoking and the prevalence of breastfeeding. Smoking among breastfeeding women was associated with shorter duration and lower milk production [43,44,45,46,47]. In the current study, however, differences were found regarding smoking habits for the different periods of breastfeeding, with a higher percentage of smoker women in the group that breastfed up to 12 months (35.7% of the total women in this group), and in the group that breastfed up to 6 months, the percentage of smokers (30.8% of the total number of women in this group) was higher than in the group that did not breastfeed (10.6% of the total number of women in this group). This may be because many women who quit smoking during pregnancy resumed smoking within the first 6 months after giving birth [48,49]. However, when the subsequent association analysis was currently done, no significant association between smoking and breastfeeding prevalence in the periods studied was observed.

The “Baby-friendly Hospital Initiative” [39,40,41,42] recommends that mothers should be supported and encouraged to initiate breastfeeding in the first hour after birth (step 4), and that babies and mothers should stay together 24 h a day (step 7). A delay in the initiation of breastfeeding can result in a reduced sucking capacity and receptivity of the newborn, which can result in a reduced or insufficient milk supply [42,50]. In the current study, the highest percentage of women who breastfed during the first hour postpartum was observed in the groups that breastfed until 6 or 12 months, with the prevalence of breastfeeding in the first hour postpartum being very low in the group of women who did not continue breastfeeding (“no breastfeeding” group), with significant differences between groups. However, the subsequent association analysis did not show a significant association between the prevalence of breastfeeding and breastfeeding in the first hour postpartum.

The current cohort did not show differences between the periods of breastfeeding in terms of the educational level of the woman; however, significant differences were observed between these groups in terms of the couple’s educational level and the type of caregiver, observing that the highest percentage of women that breastfed up to 12 months (six caregivers) was that whose caregiver was the partner. Among these six subjects, four had higher education. These results can be explained, in part, due to the current maternal education factor, since couples with higher levels of education who attended the sessions of the applied program may have greater control over their schedule or work environment; aspects that can provide the necessary support to breastfeed for a longer time [51]. However, the subsequent association analysis did not yield significant results.

Further studies on breastfeeding should be needed to assess the factors that influence its prevalence, since these factors can be dynamic, as well as it could appear others not previously considered.

## 5. Strengths and Limitations

The current study represents an advance in the knowledge of the prevalence of breastfeeding in terms of the interaction between health education programs and the presence of the informal caregiver as a differentiating element. This interaction can influence the acquisition of eating habits by pregnant and lactating women, to establish successful breastfeeding. However, other factors potentially important for the prevalence of breastfeeding, such as the duration of maternity leave or reductions in working hours to be able to breastfeed, were not analyzed yet. 

On the other hand, biochemical parameters potentially important in lactation such as calcium, lipid profile, or proteins were not analyzed.

In the current study, the child was followed until the child reached 12 months of age, so we do not have data on breastfeeding beyond this period. Thus, this aspect may constitute a limitation, considering the current recommendations on breastfeeding.

## 6. Conclusions

The prevalence of breastfeeding up to 12 months, in a cohort of Spanish primiparous pregnant women, increased the higher increase in vitamin B_12_ levels after applying an educational intervention program to promote breastfeeding. The prevalence of breastfeeding up to 12 months also increased with the presence of the informal caregiver in young women.

## Figures and Tables

**Table 1:** Baseline sociodemographic characteristics according to breastfeeding categories.

	No Breastfeeding † (n = 154)	Breastfeeding Up to 6 Months † (n = 42)	Breastfeeding Up to 12 Months † (n = 92)	*p*-Value *
	Median (IR)	Median (IR)	Median (IR)	
Basal age (years)	33 (8)	30 (9)	27.5 (6)	0.029
Baseline BMI (kg/m^2^)	23.1 (5.8)	22.3 (4.3)	22.9 (6)	0.851
Postpartum BMI (kg/m^2^)	25.1 (6.3)	23.7 (3.8)	24.7 (5.2)	0.817
	n (%)	n (%)	n (%)	
Nationality				
Spanish/European	125 (80.9)	32 (76.9)	59 (64.3)	0.622
South American	20 (12.8)	7 (15.4)	23 (25.0)	
Others	10 (6.4)	3 (7.7)	10 (10.7)	
Educational level				
Primary	7 (4.3)	10 (23.1)	23 (25)	0.074
Secondary	85 (55.3)	13 (30.8)	39 (42.9)	
Higher education	62 (40.4)	19 (46.2)	30 (32.1)	
Work ‡	111 (73.9)	32 (76.9)	76 (79.3)	0.865
Partner ‡	151 (97.9)	36 (84.6)	89 (96.4)	0.122
Educational level partner				
Primary	0 (0.0)	0 (0.0)	20 (22.2)	0.004
Secondary	88 (58.7)	23 (63.6)	30 (33.3)	
Higher education	62 (41.3)	13 (36.4)	39 (44.4)	
Contraceptives ‡	102 (66.0)	29 (69.2)	69 (75.0)	0.714
Smoking habits ‡	16 (10.6)	13 (30.8)	33 (35.7)	0.026
Alcohol consumption ‡	23 (14.9)	0 (0.0)	13 (14.3)	0.355
Family history of breastfeeding ‡	125 (80.9)	36 (84.6)	59 (64.3)	0.197
Food supplements ‡	75 (48.9)	16 (38.5)	53 (57.1)	0.526
Informal caregiver ‡	29 (19.1)	26 (61.5)	89 (96.4)	<0.001
Caregiver				
None	125 (80.9)	16 (38.5)	3 (3.6)	<0.001
Mother	23 (14.9)	16 (38.5)	66 (71.4)	
Partner	6 (4.3)	10 (23.1)	20 (21.4)	
Sister	0 (0.0)	0 (0.0)	3 (3.6)	
Breastfeeding first hour postpartum	3 (2.1)	42 (100.0)	92 (100.0)	<0.001

Abbreviations: BMI: body mass index. IR: interquartile range. † Breastfeeding categories: (1) no breastfeeding; (2) breastfeeding up to 6 months; (3) breastfeeding up to 12 months. Differences between groups for the continuous variables were tested by Kruskal–Wallis test (grouping variable: breastfeeding categories) and Games–Howel post hoc. Differences in prevalence across group were examined using χ^2^. * Results were considered statistically significant for *p*-values < 0.05. ‡ Prevalence rates for the “YES” response are shown.

**Table 2:** Biochemical parameters and body weight according to breastfeeding categories.

		No Breastfeeding † (n = 154)	Breastfeeding Up to 6 Months † (n = 42)	Breastfeeding Up to 12 Months † (n = 92)	*p*-Value
		Median (IR)	Median (IR)	Median (IR)	
Prolactin (ng/mL)	Preintervention	55.6 (64.0) ^b^	45.5 (42.1)	45.7 (16.9) ^b^	<0.001 *; 0.257 **
	Postintervention	102 (64.0) ^b^	204.6 (222.6)	256.8 (3.1) ^b^	
	∆	40 (101.3) ^e^	161 (197.6)	213.2 (210.4) ^e^	0.051 ***
Folic acid (ng/mL)	Preintervention	7 (7.8)	6.8 (8.7)	5.7 (8.0)	<0.001 *; 0.454 **
	Postintervention	8.7 (9.2) ^b^	13 (15.6)	17.9 (60) ^b^	
	∆	2.6 (12.2) ^e^	9 (16.8)	11.2 (62.3) ^e^	0.026 ***
Vitamin B_12_ (pg/mL)	Preintervention	180 (64) ^a,b^	197 (66) ^a^	230 (74) ^b^	0.142 *; 0.995 **
	Postintervention	187 (60) ^a,b^	344 (378) ^a,c^	567 (420) ^a,b,c^	
	∆	0 (62.3) ^d,e^	44 (319) ^d^	356 (397) ^e^	<0.01 ***
Transferrin (mg/dL)	Preintervention	202.5 (42) ^b^	165 (74)	154 (72) ^b^	<0.001 *; 0.093 **
	Postintervention	207.5 (61) ^a,b^	287 (116) ^a^	266 (111) ^b^	
	∆	3 (51.5)	122 (200)	115 (64.5)	0.819 ***
Body weight (kg)	First trimester	63.4 (12.4)	60.9 (12.3)	62.8 (12.8)	0.227 *; 0.452 **
	Third trimester	76.5 (13.9)	73.5 (10.6)	73.5 (11.9)	
	∆^1^	3.9 (2.8)	3.6 (1.8)	4.2 (3.1)	0.707 ***
	Third trimester	76.5 (13.9)	73.5 (10.6)	73.5 (11.9)	0.813 *; 0.915 **
	Postpartum	70 (12.5)	64.6 (9.6)	66.5 (11.5)	
	∆^2^	11.2 (2.4)	12 (1.6)	11.3 (3)	0.626 ***

Abbreviations: IR: interquartile range. ∆ Change for biochemical parameters postintervention—preintervention. ∆^1^ Change for weight third trimester—first trimester. ∆^2^ Change for weight postpartum—third trimester. † Breastfeeding group: (1) no breastfeeding; (2) breastfeeding up to 6 months; (3) breastfeeding up to 12 months. Data analyzed by two-way repeated measures, Quade non-parametric ANCOVA adjusted age. Different letters indicate statistically significant differences (*p* < 0.05) between breastfeeding groups for each timeline (a, b, c), between time (Preintervention-postintervention, within the same breastfeeding group), and between breastfeeding groups for the variation over time of the studied parameter (d, e), using pairwise comparisons with Bonferroni adjustment according to the results of the Levene test and Tukey post hoc. * *p*-value multivariate test intra-subjects for time × breastfeeding group. ** *p*-value multivariate test intra-subjects for time. *** *p*-value multivariate test inter-subjects.

**Table 3:** Factors associated with breastfeeding prevalence.

	β (ES)	OR	95% CI for OR
No breastfeeding vs breastfeeding up to 6 months			
Intersection	3.493 (2.636)		
Age	−0.126 (0.81)	0.881	0.752–1.008
∆ Vitamin B_12_	0.003 (0.003)	1.003	0.997–1.008
Caregiver intervention (no caregiver)	−1.633 (0.845)	0.195	0.37–1.024
No breastfeeding vs breastfeeding up to 12 months			
Intersection	7.086 (2.804) *		
Age	−0.246 (0.91) **	0.782	0.654–0.935
∆ Vitamin B_12_	0.007 (0.003) *	1.007	1.002–1.012
Caregiver intervention (no caregiver)	−2.734 (1.024) **	0.065	0.009–0.483

Abbreviations: ∆ Change for vitamin B_12_ postintervention—preintervention. β: β coefficient. CI: confidence interval. OR: odds ratio. The association between breastfeeding groups and potential predictors was analyzed using multinomial logistic regression (final model shown). Data entry method: backward. Methods of elimination: likelihood ratio and Wald’s criterion. Dependent variable: breastfeeding groups. Factors: caregiver intervention. Covariables: age, ∆ vitamin B12 (postintervention—preintervention. R^2^ = 0.517(Cox and Snell), 0.600 (Nagelkerke). Chi-square model = 63.987, *p* < 0.001. * *p* < 0.05; ** *p* < 0.01.

## Data Availability

There are restrictions on the availability of data for this trial, due to the signed consent agreements around data sharing, which only allow access to external re-searchers for studies following the project purposes. Researchers wishing to access the trial data used in this study can make a request to pep.tur@uib.es.

## Abbreviations

BMI: Body Mass Index; National Institute of Statistics (NIS); United Nations International Children’s Emergency Fund (UNICEF); World Health Organization (WHO).
